# Full-length amelogenin influences the differentiation of human dental pulp stem cells

**DOI:** 10.1186/s13287-015-0269-9

**Published:** 2016-01-13

**Authors:** Iris Frasheri, Christina Ern, Christian Diegritz, Reinhard Hickel, Michael Hristov, Matthias Folwaczny

**Affiliations:** Department of Conservative Dentistry and Periodontology, Ludwig-Maximilian-University, Goethestraße, 70, D-80336 Munich, Germany; Institute for Cardiovascular Prevention, Ludwig-Maximilian-University, Munich, Germany

**Keywords:** Amelogenin, Cell signaling, Pulp biology, Gene expression, Stem cells, Extracellular matrix

## Abstract

**Background:**

Amelogenin is an extracellular matrix protein well known for its role in the organization and mineralization of enamel. Clinically, it is used for periodontal regeneration and, due to its finding also in predentin and intercellular spaces of dental pulp cells, it has recently been suggested for pulp capping procedures. The aim of this study was to analyse in vitro the effect of the recombinant human full-length amelogenin on the growth and differentiation of human dental pulp stem cells (hDPSCs).

**Methods:**

Human DPSCs were treated with a supplement of amelogenin at a concentration of 10 ng/ml, 100 ng/ml and 1000 ng/ml. The groups were compared to the unstimulated control in terms of cell morphology and proliferation, mineralization and gene expression for *ALP* (alkaline phosphatase), *DMP1* (dentin matrix protein-1) and *DSPP* (dentin sialophosphoprotein).

**Results:**

Amelogenin affects hDPSCs differently than PDL (periodontal ligament) cells and other cell lines. The proliferation rate at two weeks is significantly reduced in presence of the highest concentration of amelogenin as compared to the unstimulated control. hDPSCs treated with low concentrations present a downregulation of *DMP1* and *DSPP,* which is significant for DSPP (p = 0.011), but not for DMP1 (p = 0.395).

**Conclusions:**

These finding suggest that the role of full-length amelogenin is not restricted to participation in tooth structure. It influences the differentiation of hDPSC according to various concentrations and this might impair the clinical results of pulp capping.

## Background

In 1963 Eastoe identified a protein in the tooth structure, which presented a unique amino-acid composition, different from the collagenous enamel proteins known to this date [1]. He coined the name Amelogenin for it, and since then it has been considered a tissue-specific protein. In the next four decades, since it was identified, it was investigated in its role as a protein involved in the nucleation of crystallites. Now this structural role of amelogenin in tooth development has been well-established. Self-assembling into nanosphere aggregates and with high affinity for hydroxyapatite and collagen, amelogenin controls the structural organization and mineralization of enamel, being eventually replaced by apatite mineral [2, 3]. Until recently it was thought to be produced only from ameloblasts, being thus specifically present only in enamel, but in 1997 Hammerstrom showed its presence also in the apical area of developing roots. A few years later, several studies reported amelogenin mRNA and protein in mineralized tissues, in cells such as osteoblasts, osteoclasts, chondrocytes and cementoblasts [4]. In 2006 Deutsch revealed the expression of amelogenin not just in other mineralized tissues, but also in soft tissues (brain and eye) [5]. Since then, its involvement in signaling pathways has come into the limelight.

Focusing on the pulp-dentin microenvironment, Inai et al. localized amelogenin in the predentin and in the intercellular spaces of odontoblasts and dental pulp cells, using the technique of immunolabeling [6]. Thereafter it was suggested that amelogenin, penetrating toward the pulp, might play a role in the interaction between ameloblasts and odontoblasts. Although the pathway and the downstream action of these events remain unknown, its clinical application has advanced rapidly. Amelogenin has been effectively used in wound healing (Xelma®, Molnlycke Health Care, Gothenburg, Sweden) [7], and in regenerative periodontal treatment. It has also been propagated for off label use in pulp capping procedures. Moreover, in vivo studies in humans and animals have focused on enamel matrix derivatives (EMD) as pulp capping material [8, 9]. It is derived from the porcine extracellular enamel matrix and is constituted for the most part by the full-length protein, though other components such as low-density isoforms, enamelin and transforming growth factor beta (TGF-beta) are also present in the gel of propylene glycol alginate. Histological analyses in animals following pulp capping procedures evidence the formation of an irregular dentinal bridge, which appears to be more an osteodentin-like structure with cells present in the heterogeneous tissue [10].

The ability of human teeth to produce partial reparative dentin in response to different stimuli was noted before the conception of tissue engineering. In particular, calcium hydroxide has been successfully exploited in pulp capping procedures since the 1920s. This suggests that progenitor cells, present in fully developed tooth pulp, retain the ability to form functional odontoblasts. The presence of stem cells in dental pulp was first proposed by Fitzgerald [11] and demonstrated a decade later, in a pioneering study by Gronthos et al. These are mesenchymal cells which have shown the potential to differentiate toward mesenchymal lineages (osteoblasts, smooth muscle cells, adipocytes, chondrocytes).

Upon these findings, our main goal was to determine if the recombinant human full-length protein, acting as a signaling protein, has an effect on the pulp at a genetic level, in the view of possible implications in regenerative treatments. This study aims to analyze the potential role of different concentrations of the full-length amelogenin on the stem cells present in different niches within the dental pulp and its purported influence on their growth and differentiation.

## Materials and methods

### Cell culture

The human dental pulp stem cells hDPSCs were obtained from AllCells (Alameda, CA, USA). They had been characterized by the company by means of flow cytometry, revealing a positivity for surface markers CD73, CD90, CD105 and CD166, and negativity for CD34, CD45 and CD133. These cells were provided at the second passage and cultivated upon arrival until the fifth passage in minimal essential medium, α-modification (α-MEM) with GlutaMAX™ (Life Technologies, Carlsbad, CA, USA), supplemented with 10 % Fetal Bovine Serum Gold (PAA, Pasching, Austria), 100 U/mL Penicillin and 100 μg/mL Streptomicin (Sigma Aldrich, St. Louis, MO, USA). In order to eliminate the variability of hDPSCs culture, they were used in the same passage for all experiments. Cells in the fifth passage were chosen, as studies report that, at around the fifth passage, the dentinogenic markers appear to present peak levels of expression, suggesting a greater differentiation potential [12].

The hDPSCs were assigned to four different groups: a control, and three groups with a serial dilution of amelogenin. All the experiments were carried out in biological triplicates, while PCR analyses were carried out in technical triplicates. The control group was cultivated in α-MEM supplemented as mentioned above, without other additions. The same supplemented medium was used in the amelogenin groups, adding the amelogenin protein at a concentration of 10 ng/mL, 100 ng/mL and 1000 ng/mL. Three samples were included in each group.

The amelogenin was provided by Abnova Biotec GmbH (Taipei, Taiwan): it is a human recombinant protein, full-length product of the gene *Amelx* (1-191a.a.), weighting approximately 48 kDa, which primary sequence [NX_Q99217-1] is MGTWILFACLLGAAFAMPLPPHPGHPGYINFSYEVLTPLKWYQSIRPPYPSYGYEPMGGWLHHQIIPVLSQQHPPTHTLQPHHHIPVVPAQQPVIPQQPMMPVPGQHSMTPIQHHQPNLPPPAQQPYQPQPVQPQPHQPMQPQPPVHPMQPLPPQPPLPPMFPMQPLPPMLPDLTLEAWPSTDKTKREEVD.

The cells were seeded in T25 flasks (BD Falcon, San Jose, CA, USA), at a density of 2*10^4^ cells/cm^2^ and cultured in a humidified atmosphere containing 5 % CO2 at 37 °C, with the medium changed twice a week.

### Cell morphology, proliferation and viability

The specimens were examined daily under inverted light microscopy (AXIO, Zeiss, Jena, Germany). The population doubling (PD) time and viability were evaluated passaging the cells weekly, re-plating them in T25 flasks at the starting concentration of 2*10^4^ cells/cm^2^ and counting them with an automated cell analyzer (Cedex XS, Innovatis, Basel, Switzerland), using Trypan Blue staining (Gibco, Thermo Fisher Scientific, Karlsruhe, Germany) in a 1:2 dilution, according to the manufacturer’s instructions. The PD and cumulative PD were calculated at days 7, 14 and 21 using the following formula:

$$ PD = lo{g}_2\left(P/{P}_0\right) $$

P_0_ represents the number of cells seeded at the initial passage, P is the number of cells at the next passage. The doubling time (DT) was calculated as follows:

$$ DT=CT/PD $$

with CT being the time in culture.

### Flow cytometry analysis of surface antigens

Cells were detached with Accutase (Sigma-Aldrich; 5 min at 37 °C) and then incubated with the following mouse anti-human mAbs for 30 min on ice: CD73-Pacific Blue™, CD90-FITC, CD105-FITC, CD166-PE, CD34-APC, CD45-PerCP, CD117-PE (all from BioLegend) and CD133-APC (Miltenyi Biotec). After two washing steps (PBS with 2 % FBS), the sample tubes were acquired on a BD FACSAria III (BD Biosciences) and at least 10.000 events were recorded. Data were analyzed with BD FACSDiva and FlowJo V10 software.

### Immunofluorescence analysis

hDPSC were cultured on glass slides and fixed with methanol (Carl Roth, Karlsruhe, Germany) at –20 °C for 10 min. For DMP-1 and DSPP labelling, cells were washed with phosphate-buffered saline (PBS) with Tween-20 (Carl Roth, Karlsruhe, Germany), and blocked with 10 % horse serum for 1 h at room temperature. The cells were incubated overnight at 4 °C with the primary antibodies diluted 1:200 and 1:50 to detect respectively DMP-1 and DSPP (Santa Cruz Biotechnology, Santa Cruz, CA, USA). Secondary antibodies were AlexaFluor 488 anti-rabbit and anti-goat IgG (Invitrogen, Eugene, OR, USA) used in a dilution of 1:500. For alkaline phosphatase, cells were treated with Hyaluronidase (Sigma Aldrich, Steinheim, Germany) for 30 min. After washing with PBS with Tween the same protocol was used as for DMP-1 and DSPP. The primary antibody was diluted 1:20. The nuclear counterstaining was performed with 4’,6-diamidino-2-phenylindole (DAPI) (Invitrogen, Eugene, OR, USA). Negative controls for all antibodies were carried out on the same slide omitting the primary antibody. The staining was evaluated under fluorescence microscope LSM 510 using a digital camera, AxioCam MRc (both Carl Zeiss, Jena, Germany).

### Metabolic activity

WST-1 assays were used for determining the metabolic activity. hDPSC were seeded in 24-well plates with 3*10^3^ cells/well and cultured for 21 days with the above mentioned concentrations of amelogenin. Every 7 days WST-1 (Roche Diagnostics GmbH, Mannheim, Germany) was added according to manufacturer’s protocols and the medium of each well was transferred in a 96-well plate. The cells in the 24-well plates were washed three times with PBS and further cultured in supplemented medium. Varioskan 3.00.7 was used for spectrophotometric analyses.

### Polymerase chain reaction

hDPSCs in the fourth passage were harvested at confluence, and RNA was extracted. This was considered day 0. The cells were then seeded in T75 flasks (BD Falcon, San Jose, CA, USA) at a density of 2*10^4^ cells/cm^2^ and further cultured without passaging. After 21 days cell samples underwent a process of lysation with Trizol® Reagent (Invitrogen, Carlsbad, CA, USA). The total RNA was extracted using the RNeasy Mini Kit (Qiagen, Hilden, Germany) following the manufacturer’s protocol. To exclude contamination, RNA samples with an optical density A260/A280 ratio between 1.8 and 2.1 were used.

Hexamer primer-based reverse transcription was performed for all the samples simultaneously using the First-Strand cDNA Synthesis Kit (Invitrogen, Carlsbad, CA, USA). For each sample, cDNA was synthetized using 500 ng RNA /mL.

Gene expression of alkaline phosphatase (*ALP*), dentin matrix protein-1 (*DMP1*) and dentin sialophosphoprotein (*DSPP*) was detected using a semiquantitative PCR method. Normalisation was performed using glyceraldehyde-6-phosphate dehydrogenase (*GAPDH*) as housekeeping gene. The master mix was prepared using FastStart DNA Master PLUS SYBR Green I dye.

The primers GAPDH and ALP were provided by TIB-MOLBIOL (Berlin, Germany). The primers DMP1 and DSPP for the amplification of cDNA were designed according to the published sequences, and provided by Biomol GmbH (Hamburg, Germany). The primer sequences and the thermocycling conditions are listed in Table 1.

**Table 1:** Primer sequences and thermocycling conditions used for target cDNA

Primer	Length (bp)	Sequence 5′-3′	Annealing conditions for PCR
GAPDH	238	Forward: GAG TCA ACG GAT TTG GTC GT	60 °C 30 sec; × 45cy
		Reverse: TTG ATT TTG GAG GGA TCT CG	
ALP	196	Forward: CCA CGT CTT CAC ATT TGG TG	65 °C 30 sec; × 40cy
		Reverse: AGA CTG CGC CTG GTA GTT GT	
DMP1	235	Forward: ACA GCA GCT CAG CAG AGA GT	60 °C 30 sec; × 40cy
		Reverse: TAA TAG CCG TCT TGG CAG TC	
DSPP	237	Forward: GTC GCT GTT GTC CAA GAA GA	60 °C 30 sec; × 40cy
		Reverse: ATC CTC ATC TGC TCC ATT CC	

The samples of the obtained cDNA for the two latter primers were sequenced after the gel electrophoresis run, confirming the sequence of the genes. In order to avoid variability, the samples from all the groups in the study were processed concurrently for each analysed gene. To separate the cDNA fragments obtained from the PCR, they were loaded into agarose gel with ethidium bromide staining. The primers used were of maximum 238 bp, so a 1.5 % agarose gel in Tris-Borate EDTA (TBE) buffer and GeneRuler Low Range DNA Ladder 25 to 700 bp (Thermo Scientific) were used. The PCR products were then separated by electrophoresis at 70 mV and visualized under ultraviolet light. InfinityCapt software (Vilber Lourmat, Marne-la-Valée, France) was used as a gel documentation and quantification system. This represents a limitation of our study. We are aware that our data are semiquantitative and treat them as such.

### Alizarin red staining

The capacity of the full-length amelogenin to induce the formation of mineral nodules was evaluated using Alizarin Red Staining (Sigma-Aldrich-A5533). In order to detect the alleged role as an inducer, the hDPSCs were plated in 12-well plates at a concentration of 10^4^ cells/well and cultured for 21 days with the same media as described above. The respective media were replaced every 3–4 days. Staining protocol as described by Prockop et al. was used. Briefly, the wells were washed three times with PBS, fixed in 70 % Ethanol at –20 °C for 1 h. They were subsequently washed with H_2_O, stained for 10 min with Alizarin Red, washed again with PBS and kept at a temperature of 4 °C.

### Statistical analyses

Statistical analyses of quantitative data were performed using SPSS Software Program (version 22, SPSS Inc, Chicago, IL, USA). All datasets have been tested for normal distribution using the Kolmogoroff-Smirnov test or Shapiro-Wilk test and for homogeneity of variances using Levene test. Comparisons between different stimulation conditions have been performed using Wilcoxon test or Games-Howell test. Where appropriate (comparisons between two groups) all test procedures were two-tailed. *P* values <0.05 have been considered significant.

## Results

### Phenotypic expression

#### Cell morphology and proliferation

**Fig. 1:**
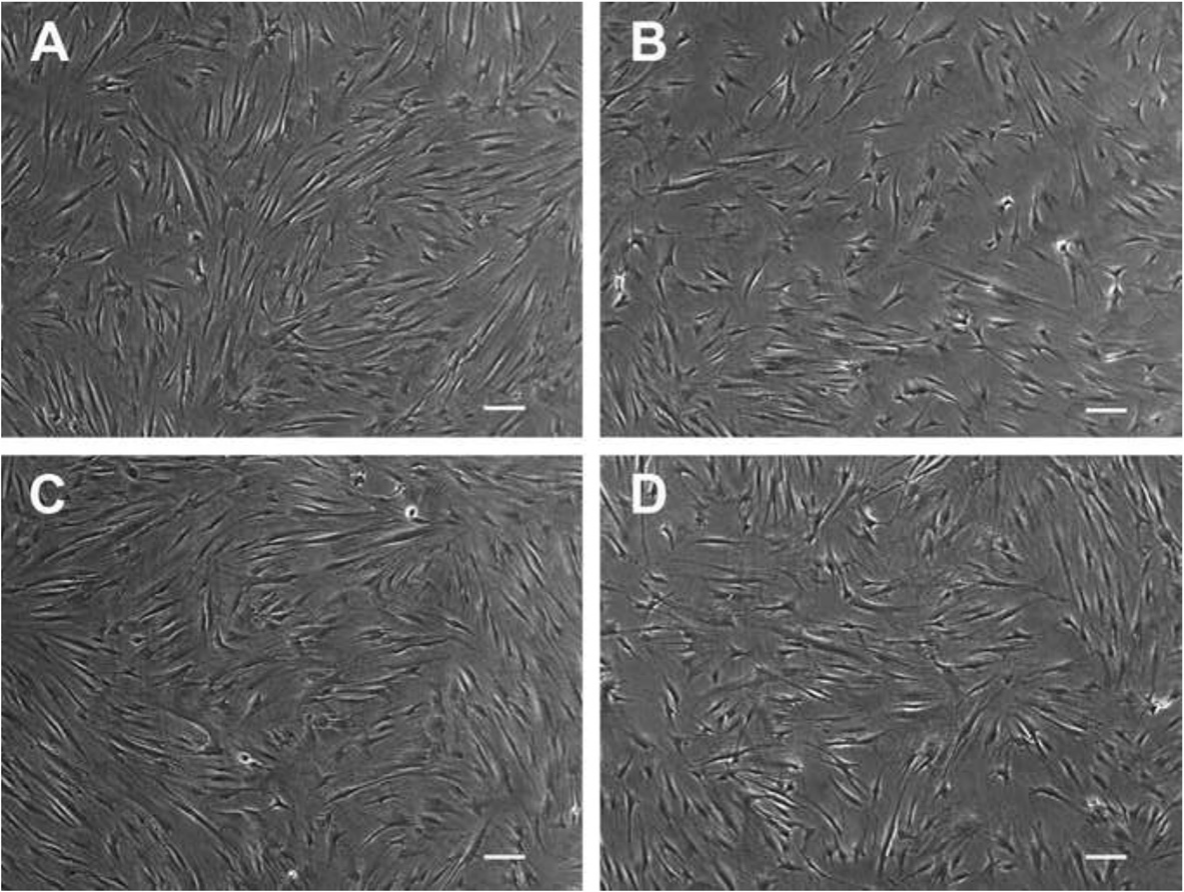
Representative light microscopy images of human dental pulp stem cells (hDPSCs). The cells were seeded in T25 flasks at a density of 2*10^4^cells/cm^2^ and cultured in minimal essential medium, α-modification supplemented with 10 % fetal bovine serum and 1 % Penicillin/Streptomicin and monitored at day 21 (×10) (**a**); hDPSCs after 21 days of cultivation with a supplement of 10 ng/mL (**b**), 100 ng/mL (**c**) and 1000 ng/mL amelogenin (**d**). *Scale bars* 100 μm

The monitoring of morphological changes in response to different amelogenin concentrations revealed no substantial differences between the control and the stimulated groups. The cells presented a spindle shape and conserved a high nucleus:cytoplasm ratio 1:2 and prominent nucleoli. Attention was also given to the pattern formation as a differentiation index of the cells, as lately published [13]. In all the flasks the plated cells were capable of forming a herringbone pattern at ×5 view, with characteristic parallel arrays observed under a magnification of ×10 and ×20 (Fig. 1). These characteristics were constant in all the groups at all time points.

**Fig. 2:**
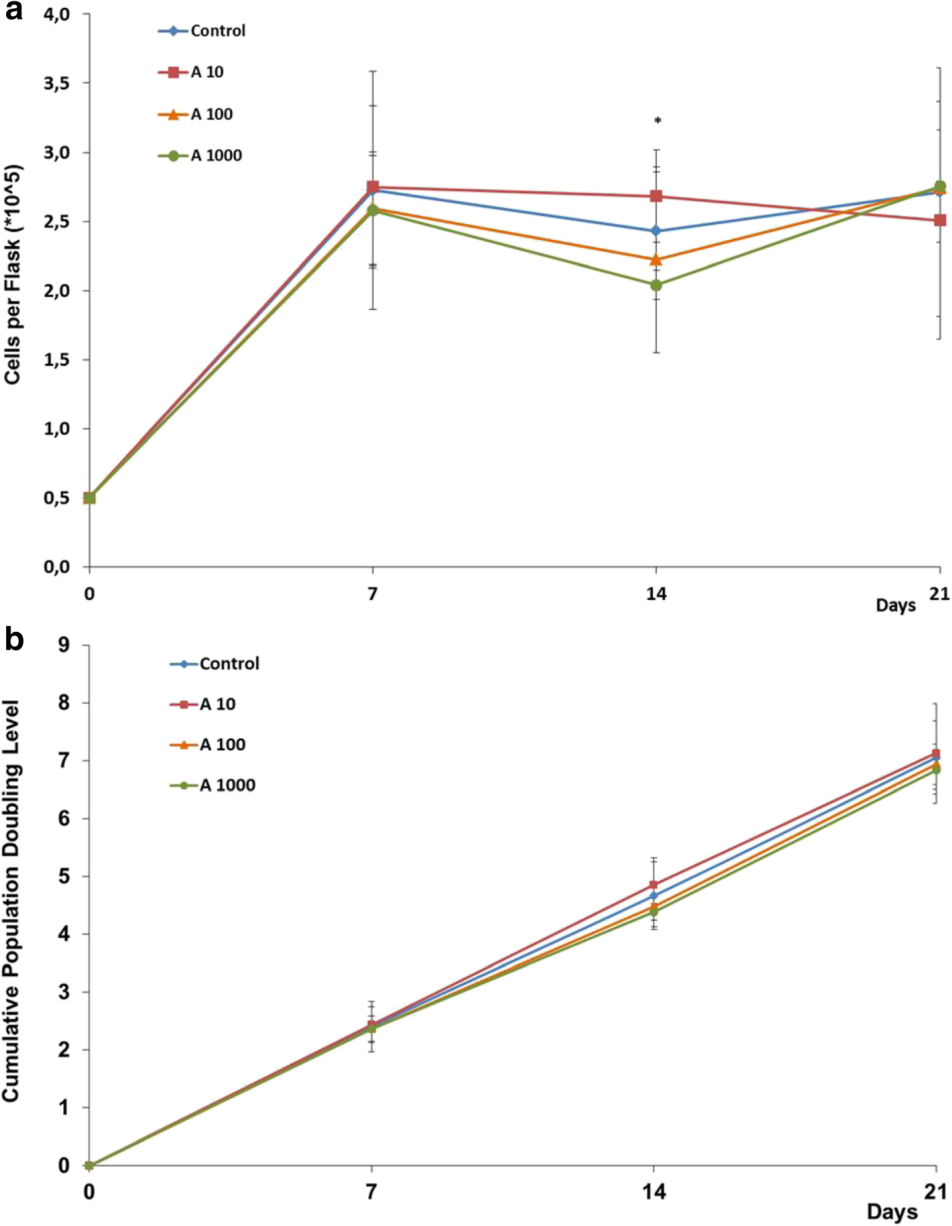
Growth curve (**a**) and cumulative population doubling levels (**b**) of human dental pulp stem cells supplemented with different amelogenin concentrations. A10, 10 ng/mL; A100, 100 ng/mL; A1000, 1000 ng/mL amelogenin; or without amelogenin supplement (control) (means ± standard deviation). *Significant differences, *p* <0.05

From the results we obtained regarding proliferation, the full-length amelogenin does not seem to significantly affect the proliferation rate of this dental pulp cell line (*p* <0.05) (Fig. 2).

The exposure of cells to 10 ng/mL human full-length amelogenin resulted in a slight increase of the growth rate (10 % compared to the control, *p* = 0.814) during the second week of cultivation. The exposure of cells to 100 and 1000 ng/mL human full-length amelogenin resulted, on the contrary, in a slight decrease of the growth rate (8 %; *p* = 0.195 and 16 %; *p* = 0.014 respectively, compared to the control) during the same week. Presence of higher concentrations of amelogenin has significantly reduced the proliferation rate. Also the difference in proliferation as observed for stimulation with 10 ng/mL vs. 1000 ng/mL was shown to be significantly different. There was no statistically significant difference in the cumulative PD (Fig. 2b), which indicated a constant trend during the three weeks. Analysis on cell viability showed a significant decrease after a stimulation with 10 ng/mL and 100 ng/mL compared to the control and to 1000 ng/mL, particularly at day 7 (*p* = 0.028) and 14 (*p* = 0.046).

#### Characterization of hDPSCs by flow cytometry

**Fig. 3:**
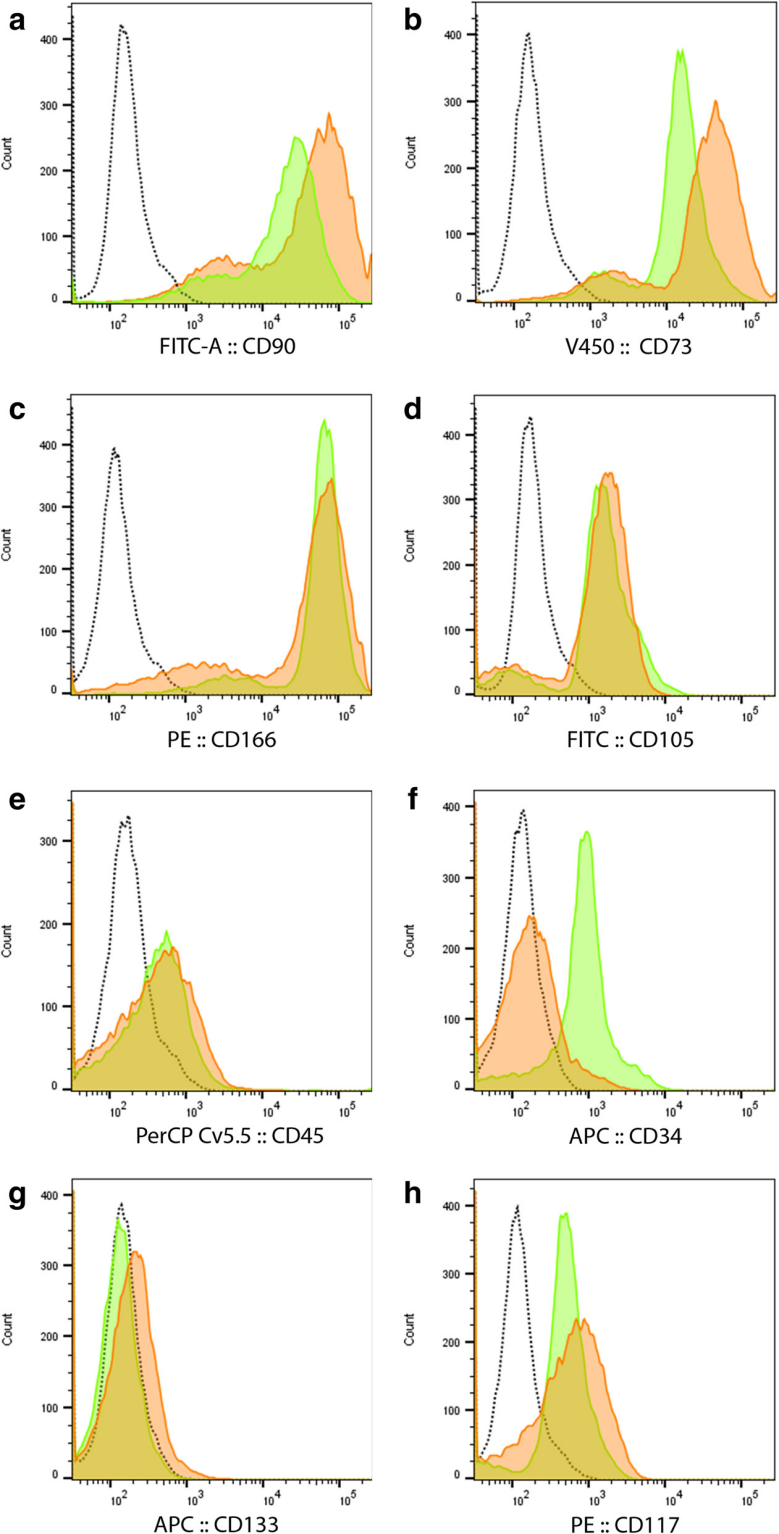
Representative histogram overlays of CD markers as analyzed by flow cytometry: CD90 (**a**), CD73 (**b**), CD166 (**c**), CD105 (**d**), CD45 (**e**), CD34 (**f**), CD133 (**g**), CD117 (**h**). *Dotted line* unstained control; *green* day 0; *orange* day 21

Freshly cultured (day 0) hDPSCs showed high expression of CD90, CD73, CD166 and CD105 (Fig. 3a, b, c, d). These cells also express CD34, some CD117 and CD45 but are negative for CD133. After 21 days in culture the hDPSCs still maintained high expression of CD90, CD73, CD166 and CD105 but were no longer positive for CD34 and had lower expression of CD45, while the expression levels of CD133, CD117 remained similar to day 0 (Fig. 3). No variability was evidenced between the study groups.

#### Immunofluorescence analysis

**Fig. 4:**
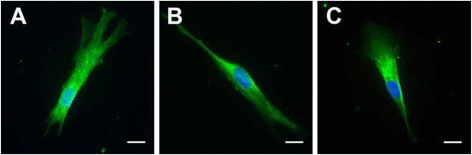
Immunofluorescence assay for dentin matrix protein-1 (**a**), dentin sialophosphoprotein (**b**) and alkaline phosphatase (**c**). Representative fluorescence microscopy photographs of human dental pulp stem cells stained with respective primary antibodies after 21 days in culture. Nuclei were stained with 4’,6-diamidino-2-phenylindole (*blue*). *Scale bars* 20 μm

Immunofluorescence staining showed a relatively homogeneous pattern of protein labeling in different cells of the same hDPSC population. Labeling for DMP1 and ALP revealed a fibrillary intracellular pattern relatively homogeneous throughout the whole cytoplasm (Fig. 4a and c), while assuming a more granular appearance for DSPP (Fig. 4b). Positive reactions to all antibodies tested were observed irrespective of the group analyzed.

### Metabolic activity

**Fig. 5:**
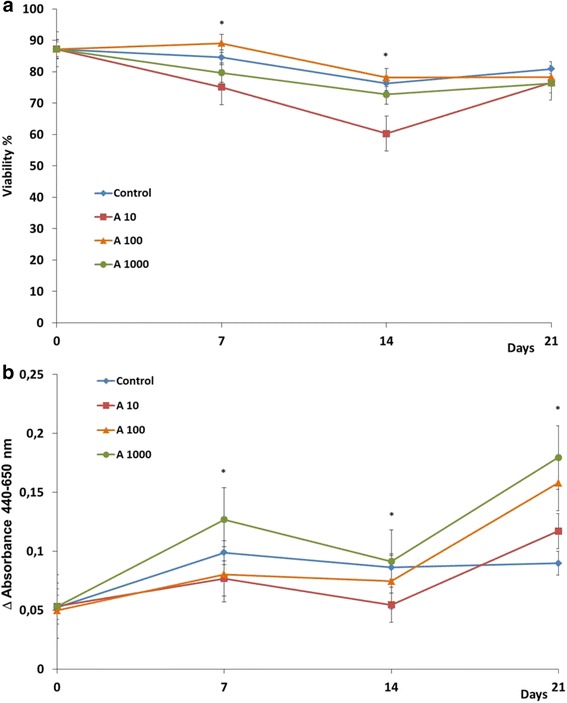
Viability (**a**) and Tetrazolium dye reduction (**b**) by human human dental pulp stem cells in the presence of different amelogenin concentrations. *A10* 10 ng/mL; *A100* 100 ng/mL; *A1000* 1000 ng/mL amelogenin; medium without amelogenin supplement (Control) (means ± standard deviation). *P <0.05

The WST-1 assays (Fig. 5b) showed the conversion of tetrazolium salts in formazan products to be lower in the presence of lower quantities of amelogenin 10 and 100 ng/mL, compared to the control. This constant trend during the first 14 days swaps at day 21. The low viability of the cells stimulated with 10 ng/mL amelogenin is associated with a lower metabolism of tetrazolium.

### Alizarin red staining

**Fig. 6:**
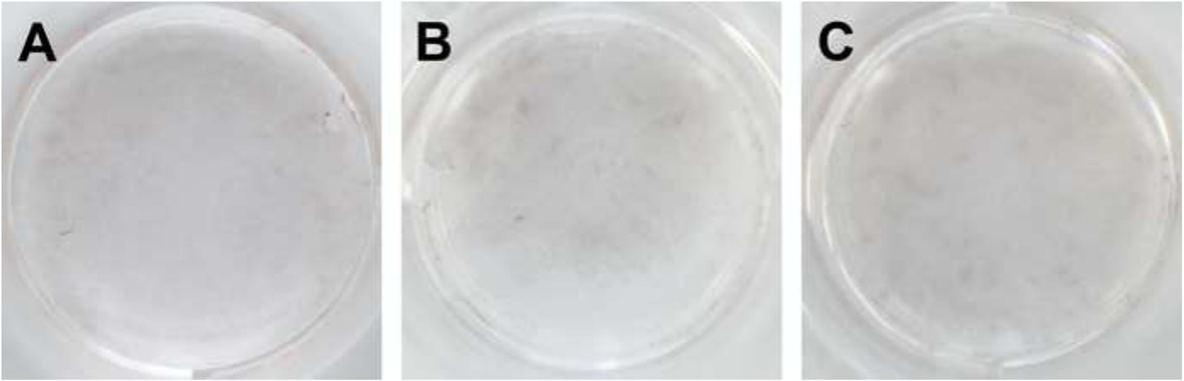
Alizarin Red staining. Human dental pulp stem cells seeded in 12-well plates at a concentration of 10^4^ cells/well and cultured for 21 days without amelogenin supplement (**a**, control) and with 10 (**b**) or 100 ng/mL (**c**) full-length amelogenin

During 21 days, the cells formed a tightly packed monolayer culture but without deposition of mineral aggregates, as seen in the control and in the amelogenin groups, regardless of the concentration of supplement used. There was no addition of any osteogenic media, and amelogenin alone does not seem to be sufficient to elicit mineral nodule formation (Fig. 6).

### Effect of rhAmelogenin on odontogenic gene expression

**Fig. 7:**
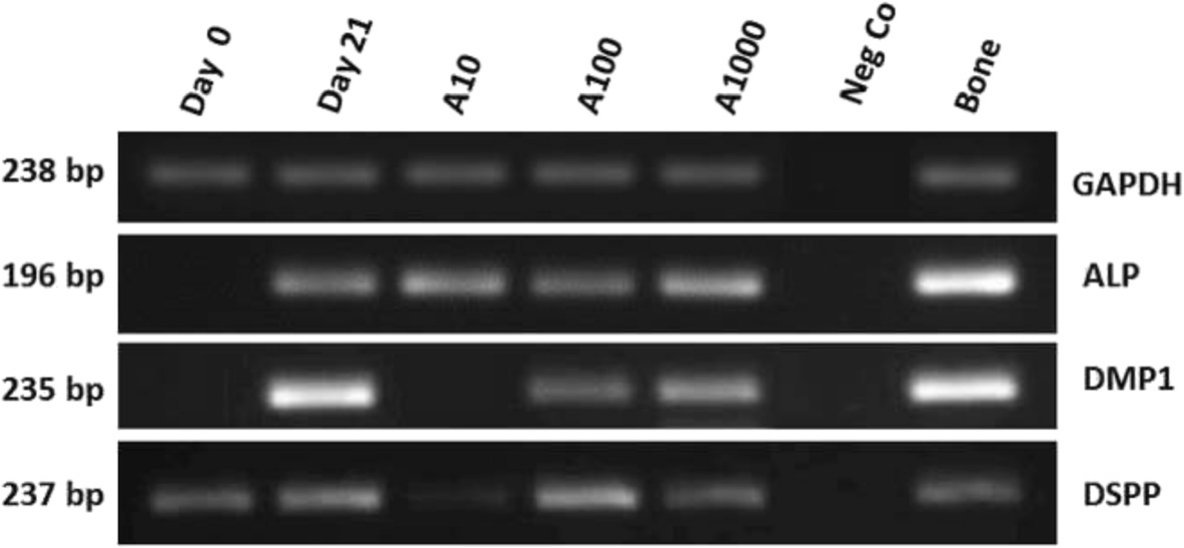
Expressions of alkaline phosphatase (*ALP*), dentin matrix protein-1 (*DMP1*), dentin sialophosphoprotein (*DSPP*) in human dental pulp stem cells (hDPSCs). Total RNAs were extracted and reversely transcribed. The cDNAs were amplified by PCR using the primers shown in Table 1. The PCR products were run on 1.5 % agarose gel and stained with ethidium bromide. *Lane 1* hDPSC expression at the initial state; *lane 2* after 21-day cultivation in medium without amelogenin supplements; *lane 3* after 21 days with 10 ng/mL amelogenin supplement in the medium; *lane 4* after 21 days with 100 ng/mL amelogenin supplement in the medium; *lane 5* after 21 days with 1000 ng/mL amelogenin supplement in the medium; *lane 6* negative control PCR run without cDNA; *lane 7* positive control PCR run with bone cDNA. Each gene expression is the result of a different gel electrophoresis

At PCR analyses, hDPSCs in the control group show, after 21 day in culture, a significant upregulation of all tested differentiation marker genes, i.e., *ALP* (mature osteoblast/odontoblast marker), *DMP-1* and *DSPP* (odontoblast markers) (Fig. 7).

**Fig. 8:**
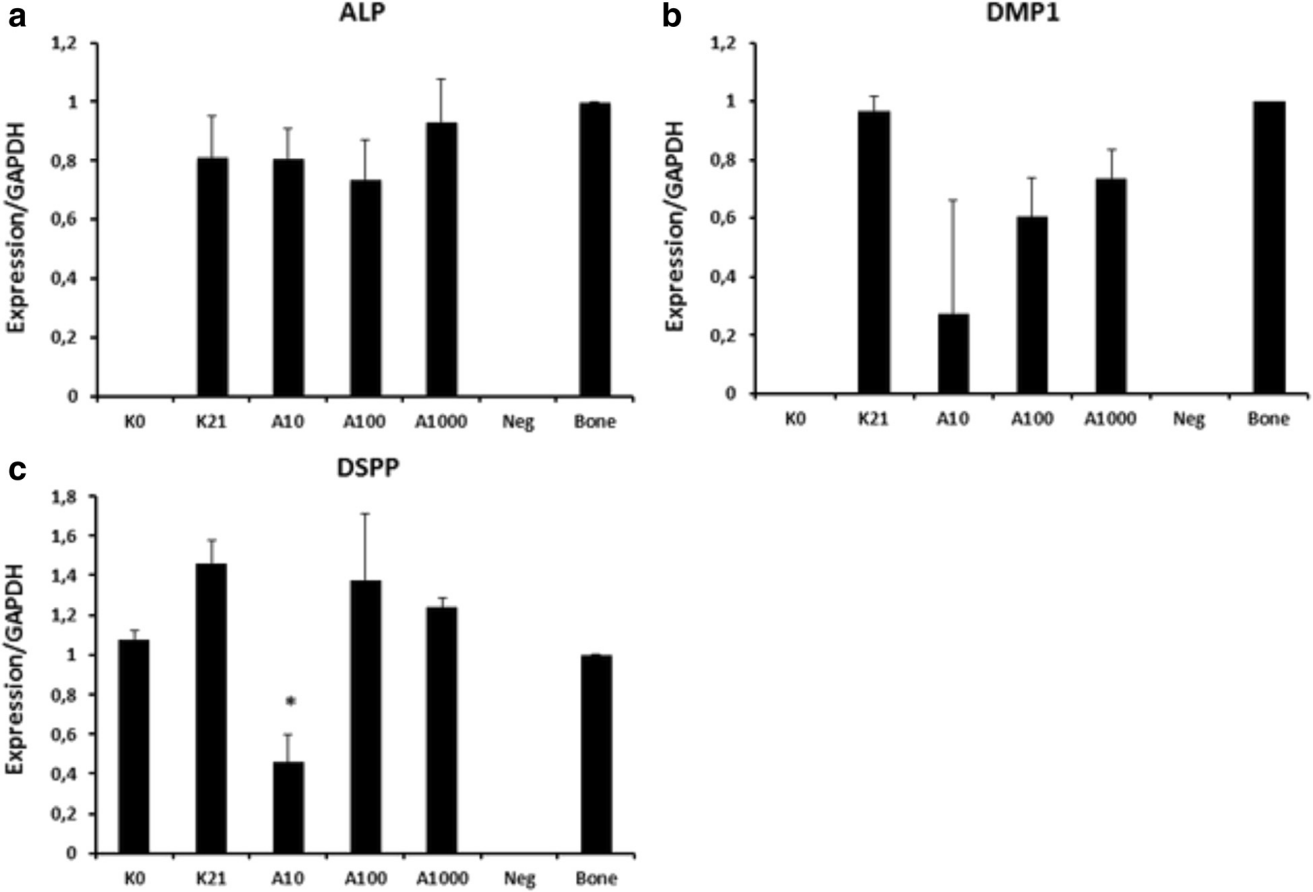
Relative expression patterns of alkaline phosphatase (*ALP*) (**a**), dentin matrix protein-1 (*DMP1*) (**b**) and dentin sialophosphoprotein (*DSPP*) (**c**) in human dental pulp stem cells (hDPSCs). Gene expression in hDPSCs before (*K0*) and after (*A10*, *A100*, *A1000*) cultivation for 21 days with amelogenin supplement of 10 ng/mL (A10), 100 ng/mL (A100), 1000 ng/mL (A1000). Control group of hDPSCs after 21 days cultivated in amelogenin-free medium (*K21*). Means ± standard deviations; *significant difference at *p* <0.05. GAPDH glyceraldehyde-6-phosphate dehydrogenase

In the 3-week-prolonged culture conditions there was a tendency for confluent growth of the hDPSCs, with a presumable increase of the cell-cell adhesion forces, thus explaining the observed response in DNA expression (Figs. 7 and 8, day 0 versus control day 21).

The expression of *ALP* at day 21 was relatively similar for all four groups. The full-length amelogenin had little influence on the gene expression, even at the highest concentration tested (1000 ng/mL), (*p* = 0.938) (Fig. 7, lanes 2–4).

Both, the expressions of *DMP1* and *DSPP* were reduced in the presence of the lowest concentration of amelogenin (10 ng/mL) compared to the unstimulated control group. However, this difference was significant for *DSPP* (*p* = 0.011) but not for *DMP1* (*p* = 0.395). The quantification of the results shows a downregulation that is inversely related to the concentration of amelogenin (Fig. 8).

## Discussion

The effect of EMD has been analyzed in different studies, with contrasting results [14]. Therefore, it is difficult to compare them and to define a uni-directional function of EMDs. First of all, there is variability in the cell line used, regarding the species (human, rat, pig, etc) and the cell type (periodontal ligament cells (PDL), bone marrow-mesenchymal stem cells (BM-MSC), gingival cells, odontoblasts, etc.). Amelogenin presents a sequence that is highly conserved among species [15–19]; therefore, the differences should not be due to the species studied, but rather the cell type seems more important. Another variable that has more influence is the isotype of amelogenin used. The latest studies show that different isotypes have different functions [20–22], so we should not refer to the role of amelogenin in general, but to the role of each specific isoform. Moreover, different concentrations are reported to be used ranging from 10 ng/mL to 100 μg/mL. EMD components have been isolated from the tooth germs in their secretory phase, and used at a concentration of 1–100 μg/mL [23]. In any case, being composed of a mixture of several enamel proteins and amelogenin isotypes, the composition is likely to vary qualitatively as well as quantitatively. In vitro studies like those of Huang [24], Izumikawa [25], and Ye [21], which have focused on single isotypes, have actually seen a greater effect on MSC and DPC at an optimal concentration of 100 ng/mL. This is also why we chose the tested concentrations, and this also permits comparability of the results with these studies. Our results on the influence of full-length amelogenin on cell morphology and proliferation of hDPSCs (Figs. 1 and 2) are in line with the findings of Izumikawa [25] who tested the full-length amelogenin on rat mesenchymal stem cells (MSC), showing similitude of this isotype activity in both species.

In our study, human amelogenin did not induce the formation of mineralized nodules in hDPSCs (Fig. 6). Our results support the findings of Ye [21] and Tanimoto [26] who reported no mineralizing effect of the full-length amelogenin on human dental papilla, dental pulp cells or PDL cells. It should be mentioned that in both studies, it was not used an osteogenic supplement. This indicates that amelogenin cannot act as an inducer of mineralization. On the other hand, studies performed using amelogenin as a supplement of osteogenic medium, show its capacity to enhance the osteogenic differentiation of BM-MSC [27], PDL cells or cementoblasts [26].

Even though amelogenin does not seem to be an inducer, the investigation of its role as an enhancer of the osteogenic differentiation and mineralization process might be of interest. Moreover, the role of low weight amelogenins, which are other important components of EMD, in mineralization has widely been confirmed [28], and they might be responsible for the clinical effect of EMD.

Regarding the ‘self-differentiation’ of this stem cell line (Figs. 7 and 8, day 0 vs. day 21), it might be due to the promoting role of fetal bovine serum supplement as suggested by Lindroos in 2009 [29]. In fact, STRO-1+ hDPSCs have been shown to consist of several interrelated subpopulations which can spontaneously differentiate into odontoblasts, osteoblasts, and chondrocytes [30]. From our FACS results, this corresponds to a selection of a subpopulation of hDPSC CD34- or to a loss of expression of CD34. As reviewed by Sidney et al. [31], there is increasing evidence that this is a marker not specifically linked to hematopoietic origin; it is also present on progenitor cells of mesenchymal cell lines and its loss suggests lineage commitment. The expression of different CD marker phenotypes correspond to a different protein setting. In concordance with our results, Yu et al. observed increased gene expression of *ALP, DSPP* and other osteoblast differentiation markers, concluding that we do not have yet an optimal medium to avoid spontaneous cell differentiation, while permitting their amplification [32]. Another possible theory, which does not exclude the first, involves mechanical reasons: the cytoskeletal tension related to cell adhesion and extracellular forces have been shown to influence stem cell (SC) commitment [33, 34]. A remarkable finding is the expression of odontoblast-related genes, *DMP1* and *DSPP* in bone, which we used afterwards as a positive control. Until now *DMP1* and *DSPP* have been considered as odontoblasts markers. *DMP1* has already been detected in osteocytes [35]. We confirm these findings for *DMP1*, and for the first time proved that *DSPP* is also expressed in bone. This means that we in fact, do not have a specific marker for odontoblasts. There is a need for further investigations into the diversification between osteoblasts and odontoblasts and how this impacts pulp regeneration therapies.

From our results, low concentrations of amelogenin determine a lower expression of odontoblast differentiation genes (Figs. 7 and 8). This is suggestive of maintenance of an undifferentiated status of the cells, similar to the starting cells at day 0, even preventing spontaneous differentiation visible in the control cultures. The higher effectiveness of lower concentrations of the protein can be explained by its three-dimensional structure and its capacity to assembly into nanospheres as described by Fang in 2011 [36]. In particular, the full-length protein has an internal structure constituted of dodecamers, not present in other isotypes. Furthermore, it is widely known that self-assembly is a common property of many extracellular organic matrix macromolecules [37], facilitated by intermolecular interactions and specifically assembly of amelogenins into nanosphere increases at increasing concentrations.

We explained the results with the presence of the protein in a monomeric state at lower concentrations, having thus a higher impact on cell signaling. On the contrary an increasing assembly into nanospheres at higher concentrations leads to its structural role. Our results support the previously reported role of amelogenin as a signaling molecule, adding the hDPSCs as another target of this protein.

Other studies of PDL cells, report a proliferative effect of EMD, but lower than that of epidermal growth factor (EGF) and platelet-derived growth factor [38, 39]. Another study by Huang et al. [24] reports a proliferative effect of the full-length amelogenin on BM-MSC, but in this case the isoform used is the rh174. Even though amelogenin represents the main component of EMD, we think that these positive results might be due to the presence of other growth factors that are components of EMD. The different results reported in different studies may be explainable with the use of different isoforms and splicing products of amelogenin, with the use of different animal or human sources of this protein and different cell types and also source of stem cells (embryonic [28], apical papilla, periodontal ligament). A comprehensive review focusing on this topic, specifically limited to in vitro studies, has been published by Grandin in 2012 and Bosshardt in 2008 [14, 40]. Recently a systematic review has summarized and compared the results of EMD capping in animal and in human studies [41], evidencing controversial results.

## Conclusions

While the effects of amelogenin have been largely studied with bone mesenchymal stem cells (BMSCs) and PDL cells [42], to our knowledge, this is the first in vitro study that analyzes the effect of full-length amelogenin on hDPSCs. The results obtained suggest that amelogenin affects hDPSCs differently than it does in PDL cells and other cell lines. We show that the full-length amelogenin seems to have a negative mitogenic impact on hDPSCs at higher concentrations but shows a tendency for positive mitogenic effects at lower concentrations.

This study permits a further understanding of the signaling role of the full-length amelogenin. Considering the present result on the inhibition of *DMP1* and *DSPP*, it seems plausible that amelogenin impairs the clinical results of pulp capping; hence previous positive clinical observations might have been random effects.

Further studies should focus on the molecular mechanisms behind the effects of amelogenin, taking into consideration that different isoforms have different roles, and considering also the cell type used as a target of the protein. For a possible application of amelogenin in pulp regeneration, odontoblasts and other cell types present in the pulp should also be analyzed.

## Abbreviations

*ALP*: alkaline phosphatase
BM-MSC: bone marrow-mesenchymal stem cells
bp: base pairs
DAPI: 4’,6-diamidino-2-phenylindole
*DMP1*: dentin matrix protein-1
*DSPP*: dentin sialophosphoprotein
DT: doubling time
EGF: epidermal growth factor
EMD: enamel matrix derivatives
FACS: fluorescence-activated cell sorting
*GAPDH*: glyceraldehyde-6-phosphate dehydrogenase
hDPSCs: human dental pulp stem cells
kDa: kiloDalton
PBS: phosphate-buffered saline
PD: population doubling
PDL: periodontal ligament
TGF: transforming growth factor
WST-1: water-soluble tetrazolium salt 1
α-MEM: minimal essential medium, α-modification
